# Corrigendum to “Toward reliable diabetes prediction: Innovations in data engineering and machine learning applications”

**DOI:** 10.1177/20552076251313644

**Published:** 2025-01-12

Talukder MdA, Islam MdM, Uddin MA, et al. Toward reliable diabetes prediction: Innovations in data engineering and machine learning applications. *Digit. Health* 2024;10. DOI: 10.1177/20552076241271867

In the above-mentioned article, the funding was listed as “The authors received no financial support for the research, authorship and/or publication of this article”. The details have been updated and the revised funding statement reads as:

**Funding:** The author(s) disclosed receipt of the following financial support for the research, authorship, and/or publication of this article: This work is supported by RSP2024R301, King Saud University, Saudi Arabia.

The article has been updated to reflect the correction.

